# Th9/IL-9 Profile in Human Echinococcosis: Their Involvement in Immune Response during Infection by *Echinococcus granulosus*

**DOI:** 10.1155/2014/781649

**Published:** 2014-03-30

**Authors:** Nannan Pang, Fengbo Zhang, Xiumin Ma, Zhaoxia Zhang, Hui Zhao, Yan Xin, Song Wang, Yuejie Zhu, Hao Wen, Jianbing Ding

**Affiliations:** ^1^Hematologic Disease Center and Xinjiang Uygur Autonomous Region Research Institute of Hematology, First Affiliated Hospital of Xinjiang Medical University, Urumqi, Xinjiang 830054, China; ^2^Department of Clinical Laboratory, First Affiliated Hospital of Xinjiang Medical University, Urumqi, Xinjiang 830054, China; ^3^State Key Laboratory Incubation Base of Major Diseases in Xinjiang and Xinjiang Key Laboratory of Echinococcosis, First Affiliated Hospital of Xinjiang Medical University, Urumqi, Xinjiang 830054, China; ^4^Basic Medical College of Xinjiang Medical University, Urumqi, Xinjiang 830011, China

## Abstract

Th9 cells have been reported to contribute to immune responses; however, the role of Th9 cells in *Echinococcus granulosus* infection is unknown. This study is to determine whether Th9 cells and IL-9 are involved in human *Echinococcus granulosus* infection. Compared with healthy controls (HC group), the mRNA levels of PU.1, IL-9, and GATA-3 were significantly increased in patients before therapy (CE group), as revealed by qRT-PCR. Flow cytometry analysis showed that the percentages of Th9 and Th2 cells in CE group were significantly higher. The levels of IL-9, IL-4, IL-10, and TGF-**β** in CE group were also significantly increased, as detected by CBA assay. The percentages of Th9 and Th2 cells in CE group were positively correlated. After treatments of surgery in combination with albendazole, the PU.1 and GATA-3 mRNA levels were significantly decreased in patients after therapy (PCE group) compared with CE group. The numbers of Th9 and Th2 cells and levels of IL-9, IL-4, IL-10, and TGF-**β** were also significantly decreased in PCE group. In conclusion, the ratios of Th9 cells and IL-9 levels were significantly decreased after treatment, suggesting that Th9/IL-9 may be involved in immune response induced by *Echinococcus granulosus* infection.

## 1. Introduction

Echinococcosis, also known as hydatidosis, is a serious zoonotic disease caused by infection with* Echinococcus*. Human echinococcosis include cystic echinococcosis (CE) and alveolar echinococcosis (AE), which are respectively caused by* Echinococcus granulosus* (Eg) and* Echinococcus multilocularis* (Em). The human secondary CE is caused by leak of protoscoleces when fertile cysts are accidentally ruptured, followed by development of new metacestodes. Because this disease has a long incubation period, most patients often have no obvious symptoms and develop into chronic infection [1–3].

After infecting the host,* Echinococcus *gradually gains the ability to escape from the host immune response so that it can survive in the host for a long time [4]. Researches in CE patients and secondary hydatid mouse model indicate that, during the late stage of hydatid infection, the Th2 cell humoral immunity is dominant and the imbalance of Th1/Th2 plays an important role in promoting the immunopathogenesis change of this disease, accompanied by secretion of cytokine IFN-*γ* and IL-4 [3, 5–7].

A new type of effector CD4^+^ T cells, which mainly secrete IL-9 and IL-10, is defined as the T helper 9 (Th9) cell subsets [8]. Th9 promotes allergic inflammation and is distinct from other CD4^+^ T subsets of Th1, Th2, Treg, and Th17 cells [9].

It is known that IL-9 is a Th2-type cytokine, which acts in a variety of inflammatory cells and tissue cells and plays important roles in parasitic infections [10–12] and allergic diseases, especially allergic reactions, asthma, and so on [13, 14]. Like mouse Th9 cells, human Th9 cells produce IL-9, but they cannot secrete IL-10 [15]. Further studies showed that CD4^+^ T cells can differentiate into Th9 cells in the presence of TGF-*β* and IL-4. And, TGF-*β* alone can divert the differentiation of Th2 towards the development of Th9 cells [9]. Studies by Chang et al. [16] showed that, like Th1 (T-bet), Th2 (GATA-3), Treg (Foxp3), and Th17 (ROR-*γ*t) cells, PU.1 was one of the specific transcription regulation factors for Th9 cells. PU.1 promotes Th9 cells to secrete IL-9 and plays a critical role in regulation of autoimmune diseases. The Th17 cells, by secretion of IL-17, IL-21, and tumor necrosis factor (TNF-*α*) [17, 18], have similar functions to Th9 cells in inflammation.

Our previous study found that the levels of peripheral Th17 cells and IL-17 were slightly lower in the CE patients than in health control, suggesting there was imbalance between Th17 cells and Treg cells in CE patients [19]. Therefore, the purposes of this study are as follows: (i) to explore the change of Th9 cells in the CE patients before and after treatments; (ii) to study the characteristics of the changes of Th9-related cytokines and transcription factors; (iii) to study the correlations between Th9 and Th1, Th2, Treg, and Th17 cells in CE patients.

## 2. Materials and Methods

### 2.1. Patients

The twenty-seven hepatic hydatid patients in this study were all from the First Affiliated Hospital of Xinjiang Medical University, who received treatment during December 2009 and December 2012 (Table 1). The health control group (HC group) included 25 medical staffs whose ages and sexes were matched with the patients. The CE patients were divided into two groups: (1) CE group (pretherapy group): 27 CE patients who were diagnosed by imaging examination but had not received any therapy; (2) PCE group (posttherapy group): 27 CE patients who were successfully cured by surgery in combination with albendazole. Within 6 months, no relapse and metastasis were found in these patients as examined by imaging and no serious infections and obvious symptoms were detected in PCE group. Clinical data of healthy individuals and CE patients before and after treatment were included in Table 1. The cysts were all staged according to the WHO-IWGE classification [20, 21]. This study was approved by the Human Ethics Committee of the First Affiliated Hospital of Xinjiang Medical University (Approval number: 20120220-126). All patients provided written informed consents.

### 2.2. Quantitative RT-PCR

Total RNA was extracted according to the RNA isolation kit (Qiagen, Valencia, CA, USA) instructions. Then RNA (1 *μ*g) from each sample was reverse transcribed into cDNA (Invitrogen, Carlsbad, CA, USA). All primers and annealing temperatures were shown in Table 2. The real-time RT-PCR was conducted with the SYBR Green PCR premix (TaKaRa Biotechnology (Dalian) Co., Ltd., Dalian, China) following the manufacturer's protocols. To normalize gene expression, mRNA expression of the housekeeping gene beta-actin (*β*-actin) was also measured. For each sample, both the housekeeping and the target genes were amplified in triplicate using the following procedure of initial denaturation at 95°C for 1 min, 40 cycles of 95°C for 5 s, 58°C (or other) for 30 s, and 72°C for 30 s. The 2^−ΔΔCt^ method was used to determine the cycle number Ct value corresponding to a specific fluorescence threshold and quantify the target genes.

### 2.3. Cytometric Bead Array (CBA)

The concentrations of IFN-*γ*, IL-9, IL-17, IL-4, IL-6, IL-10, and TGF-*β* in the sera were quantitatively determined by the CBA kit-BD (BD Biosciences, San Jose, CA, USA). Briefly, 50 *μ*L of serum was used in duplicate for analysis according to the manufacturer's protocol. The CBA technique was based on 7 bead populations with distinct fluorescence intensities that were coated with antibodies specific for IFN-*γ*, IL-9, IL-17, IL-4, IL-6, IL-10, and TGF-*β* proteins. Four standard curves (range from 0 to 5000 pg/mL) were obtained from one set of calibrators and four sets of results were obtained on one test sample. CBA was used to measure these cytokines on a flow cytometry (ASR II). The data were analyzed using FCAP Array software that was designed for CBA data analysis.

### 2.4. Flow Cytometry

Peripheral blood mononuclear (PBMC) was isolated by Ficoll-Hypaque density centrifugation (Amersham Biosciences, UK). PBMCs were suspended at a density of 2 × 10^6^ cells/mL in Roswell Park Memorial Institute (RPMI) media 1640 with GlutaMAX (Gibco, USA).

For analysis of Th9/Th1/Th2/Th17/Treg cells, 2 × 10^6^ PBMC cells were seeded in 24-well plates and were stimulated with phorbol myristate acetate (PMA, 50 ng/mL) plus ionomycin (1 µM, all from Alexis Biochemicals, San Diago, CA, USA) and monensin (500 ng/mL, from eBioscience, San Diago, CA, USA) for 5 hours. The cells were collected and stained with antibodies against APC-CD3 and PE-Cy5- at 4°C for 30 minutes. After surface staining, the cells were fixed and permeabilized, followed by incubation with antibodies against IL-9-PE, IFN-*γ*-PE-cy7, IL-4-PE-cy7, Foxp3-PE-cy7, and IL-17-PE-cy7 for 30 minutes. Isotype-matched controls were used to correct nonspecific binding. All of the antibodies and reagents were purchased from eBioscience (San Diego, CA, USA). In the flow cytometry, the cells were gated on the forward scattering of living cells and then centered on CD3^+^CD4^+^T cells. All stained cells were analyzed by flow cytometry (ASR II) and FlowJo software (Tristar, USA). At least 50,000 events per samples were analyzed.

### 2.5. Statistical Analyses

The SPSS13.0 software package was used for statistical analysis, and the measurement data was presented as $\overset{-}{x}\pm s$. If the data did not meet the normal distribution, they were logarithmically transformed into normal distribution. If the variances were homogeneous, one-way analysis of variance (ANOVA) was used for comparison of the averages between groups. If the variances were not homogeneous, rank sum test was used.* Spearman* rank correlation analysis was used for correlation analysis. *P* < 0.05 was considered statistically significant.

## 3. Results

### 3.1. Expression Levels of PU.1 and GATA-3 in PBMCs Are Related to Development of CE

**Figure 1:**
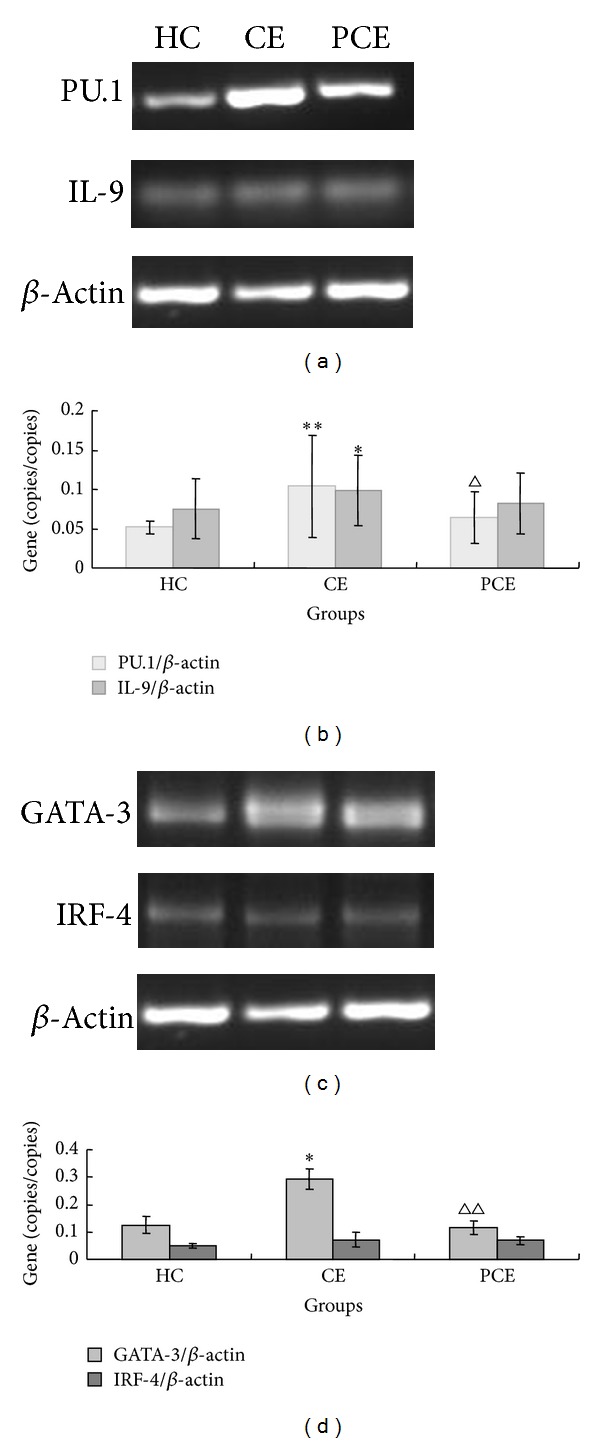
Quantitative RT-PCR. Total RNAs were extracted and subjected to reverse transcription, followed by determination by quantitative RT-PCR. The 2^−ΔΔCt^ method was used to determine the cycle number Ct value corresponding to a specific fluorescence threshold and quantify the target genes. *β*-Actin was used as an internal control. (a) The PU.1 and IL-9 mRNA expression in HC, CE, and PCE groups. (b) Relative expression values of PU.1 and IL-9 mRNA were calculated based on the values of *β*-actin. (c) The GATA-3 and IRF-4 mRNA expression in HC, CE, and PCE groups. (d) Relative expression values of GATA-3 and IRF-4 mRNA were calculated based on the values of *β*-actin. *P < 0.05; **P < 0.01, compared with the HC group. ^△^ *P* < 0.05; ^△△^ *P* < 0.01, compared with CE group. HC: healthy control; CE: CE pretherapy group; PCE: CE posttherapy group.

PU.1, GATA-3, and IRF4 are key transcription factors that induce differentiation of Th0 cells into Th9 cells. IL-9 is the main cytokine secreted by Th9 cells. To determine if PU.1, IL-9, GATA-3, and IRF4 are related to development of CE, the mRNA expression levels of PU.1, IL-9, GATA-3, and IRF4 in peripheral blood were compared in the CE, PCE, and HC groups by quantitative RT-PCR. As shown in Figures 1(a) and 1(b), the mRNA expression levels of PU.1 and IL-9 in the CE group were significantly higher than those in the HC group (PU.1, *P* = 0.000; IL-9, *P* = 0.032). PU.1 expression was significantly decreased in the PCE group when compared with the CE group (PU.1, *P* = 0.04). Meanwhile, there was no significant difference in PU.1 mRNA level between PCE group and HC group (PU.1, *P* = 0.266). As shown in Figures 1(c) and 1(d), GATA-3 mRNA level of CE group was significantly higher than that of HC group (*P* = 0.012) and PCE group (*P* = 0.009). The difference in GATA-3 mRNA level between HC group and PCE group was not significant (*P* = 0.998). The expression levels of IRF4 in CE group and PCE group were slightly higher than those in the HC group. However, the changes in IRF4 mRNA levels among HC group, CE group, and PCE group were not statistically significant (*P* > 0.05). The PU.1 and GATA-3 gene copies in the PCE group were decreased than those in the CE group, possibly due to the recovery of the patients after surgery. These results suggest that PU.1 and GATA-3 levels are related to development of CE.

### 3.2. The Th9 and Th1, Th2, Treg, and Th17 Cell Numbers in the Peripheral Blood of the Three Groups

**Figure 2:**
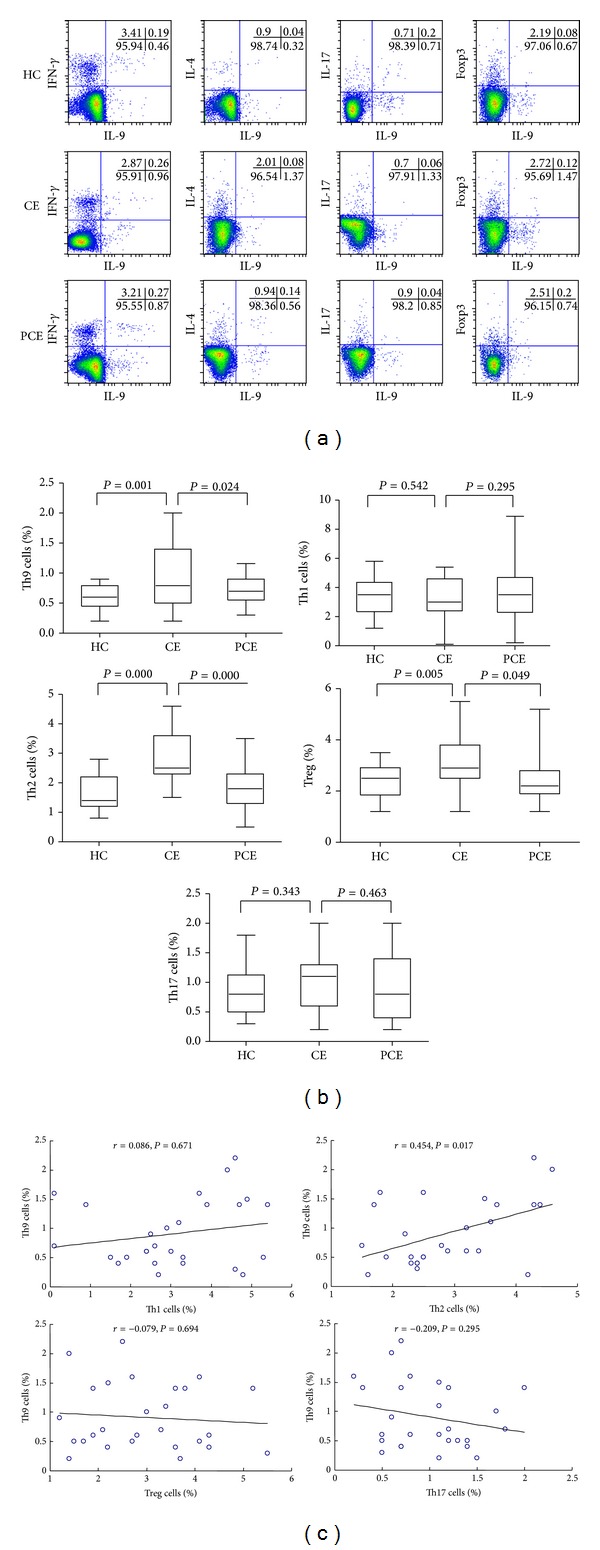
Percentages of Th9, Th1, Th2, Th17, and Tregs cells in peripheral blood. Subtypes of CD4^+^ T cells in peripheral blood were detected by flow cytometry analysis. The detected cell types included Th9 (IL-9^+^) cells, Th1 (IFN-*γ* ^+^) cells, Th2 (IL-4^+^) cells, Treg (Foxp3^+^) cells, and Th17 (IL-17^+^) cells. Cells were first gated on CD3^+^CD4^+^ T cells and then on IL-9^+^/IFN-*γ* ^+^/IL-4^+^/Foxp3^+^/IL-17^+^CD3^+^CD4^+^ T cells, respectively. (a) The representative flow cytometric dot-plots of Th9, Th1, Th2, Th17, and Tregs cells in the blood of the HC, CE, and PCE groups were shown. Numbers within each quadrant indicated percentages of cells within each dot-plot. (b) Comparisons of the percentages of Th9, Th1, Th2, Th17, and Tregs cells in the peripheral blood of the HC, CE, and PCE groups. Horizontal bars indicated means. The percentages of Th cells represented Th cell numbers in total CD4^+^ T-cell numbers were determined by flow cytometry. (c) Th9 cells were correlated negatively with Th1, Th17, and Tregs cells, while Th9 cells were positively correlated with the numbers of Th2 in peripheral blood in CE patients. Correlations were determined by Spearman rank correlation coefficients. HC: healthy control; CE: CE pretherapy group; PCE: CE post-therapy group.

Subtypes of CD4^+^ T cells in peripheral blood were detected by flow cytometry analysis. The detected cell types included Th9 (IL-9^+^) cells, Th1 (IFN-*γ* ^+^) cells, Th2 (IL-4^+^) cells, Treg (Foxp3^+^) cells, and Th17 (IL-17^+^) cells. Representative dot-plot results of flow cytometry analysis were shown Figure 2(a). Cells were first gated on CD3^+^CD4^+^ T cells and then on IL-9^+^/IFN-*γ* ^+^/IL-4^+^/Foxp3^+^/IL-17^+^CD3^+^CD4^+^ T cells, respectively. Numbers within each quadrant indicated percentages of cells within each dot-plot. Then we compared the changes of CD4^+^ T-cell subtypes among HC group, CE group, and PCE group. As shown in Figure 2(b), ratios of the circulating Th9 cells were increased in the patients before therapy but decreased after therapy. The percentage of Th9 cells (CD3^+^CD4^+^IL-9^+^/CD4^+^ T cells) in CE group was 0.91 ± 0.49%, significantly higher than that in HC group (0.6 ± 0.2%)(*P* = 0.001), while the Th9 cell ratio of the PCE group was 0.7 ± 0.21%, significantly lower than that of CE group (*P* = 0.024). The ratio of Th1 cells (CD3^+^CD4^+^IFN-*γ* ^+^/CD4^+^ T cells) in the HC group, CE group, and PCE group were 3.37 ± 1.25%, 3.09 ± 1.48%, and 3.57 ± 2.12%, respectively. The differences among CE group, PCE group, and the HC group were not statistically significant (*P* > 0.05). When compared with the HC group (1.66 ± 0.59%), the ratio of Th2 cells (CD3^+^CD4^+^IL-4^+^/CD4^+^ T cells) in CE group (2.89 ± 0.93%) was significantly increased (*P* = 0.000). Meanwhile, compared with CE group, the ratio of Th2 cells in PCE group was decreased significantly (1.79 ± 0.8%, *P* = 0.000). The ratio of Treg cells (CD3^+^CD4^+^Foxp3^+^/CD4^+^ T cells) in CE group (3.15 ± 1.03%) was also increased, compared with HC group (2.36 ± 0.65%) (*P* = 0.005). The Treg cell ratio in the PCE group (2.49 ± 0.94%) was significantly decreased compared with CE group (*P* = 0.049). The ratio of Th17 cells (CD3^+^CD4^+^IL-17^+^/CD4^+^ T cells) in HC group, CE group, and PCE group was 0.86 ± 0.4%, 0.99 ± 0.46%, and 0.89 ± 0.55%, respectively. The differences in Th17 cell ratio among these three groups were not statistically significant (*P* > 0.05).

Since previous studies report that IL-9 is also secreted by Th17 cells [22, 23] and Tregs [24] and that Th9 cells are tightly associated with Th2 cells [25], the correlations between Th9 cells and the other subsets were investigated. As shown in Figure 2(c), Th9 cell number was positively correlated with that of Th2 (*r* = 0.454, *P* = 0.017), but not with Th1 (*r* = 0.086, *P* = 0.671), Th17 (*r* = −0.209, *P* = 0.295), or Tregs (*r* = −0.079, *P* = 0.694). These results suggest that in CE patients, levels of IL-9 in the circulating blood were related to numbers of Th2 cells.

### 3.3. Alterations in Cytokine Levels in Serum of Patients

**Figure 3:**
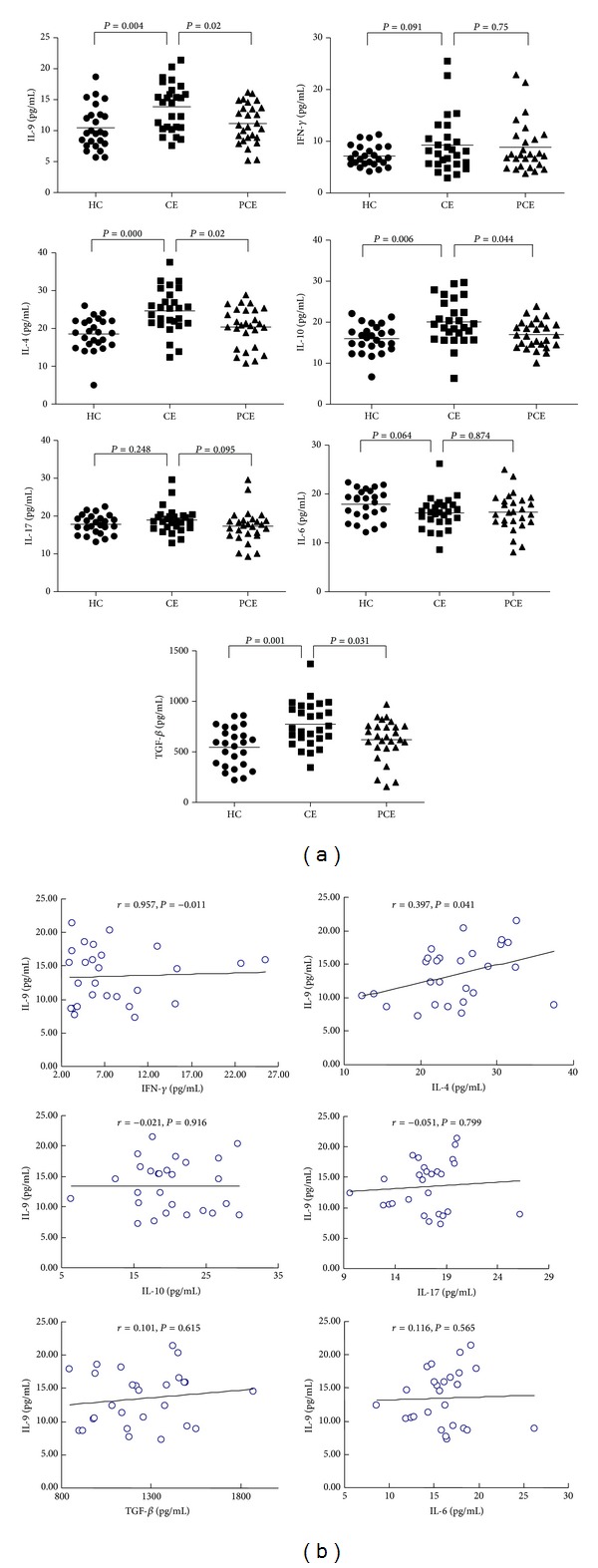
Th9 related cytokines changed in CE patients as determined by the CBA experiment. (a) Comparisons of levels of IL-9, IFN-*γ*, IL-4, IL-10, IL-17, TGF-*β*, and IL-6 in HC, CE, and PCE groups. The horizontal bars indicated means. Comparison was performed using one-way analysis of variance. (b) In CE patients, IL-9 concentrations were correlated negatively with IFN-*γ*, IL-10, IL-17, TGF-*β*, and IL-6 in CE patients (*r* = −0.011, *r* = −0.021, *r* = −0.051, *r* = 0.101, and *r* = 0.116, resp.). IL-9 was positively correlated with the concentrations of IL-4 (*P* = 0.041, *r* = 0.397).

To determine the levels of cytokines in serum of patients, CBA experiment was performed. As shown in Figure 3(a), when compared to the HC group, the levels of IL-9, IL-4, IL-10, and TGF-*β* in the serum were all significantly increased (IL-9, *P* = 0.004; IL-4, *P* = 0.000; IL-10, *P* = 0.006; TGF-*β*, *P* = 0.001) in CE group. When compared to CE group, the level of IL-9, IL-4, IL-10, and TGF-*β* was significantly decreased after successful surgery treatment in the PCE group (IL-9, *P* = 0.02; IL-4, *P* = 0.02; IL-10, *P* = 0.044; TGF-*β*, *P* = 0.031). Levels of IFN-*γ*, IL-17, and IL-6 were changed slightly in the CE and PCE groups, and the differences were not statistically significant in comparison with the HC group (*P* > 0.05). As shown in Figure 3(b), the IL-9 level in serum was positively related to IL-4 (*r* = 0.397, *P* = 0.041), but not related to IFN-*γ*, IL-10, IL-17, IL-6, or TGF-*β*. These results suggest that in the serum of CE patients, levels of multiple cytokines, including IL-9, IL-4, IL-10, TGF-*β*, IL-17, IL-6, and IFN-*γ*, were altered. Among these cytokines, levels of IL-9 were significantly increased upon infection and decreased after treatments, showing a positive correlation with level of IL-4.

## 4. Discussion

In most Eg infection, the clinical outcome is associated with activation of Th1 or Th2 cells and usually displays Th2-mediated susceptibility in the late stage of infection. It is known that chemotherapy can regulate the immune response, thus altering Th1/Th2 cytokine patterns [26]. IL-9 is thought to be a Th2 cytokine for a long time, as it promotes allergic inflammation and is associated with various Th2 responses [8, 9]. More studies suggest that IL-9 has multiple functions. Of significant importance is the recent discovery of a IL-9-producing Th subset called Th9 cells. Th9 cells play an important role in autoimmune and allergic diseases [8, 9, 15, 27–30]. However, whether Th9 cells are involved in parasite infection immunity, especially in* Echinococcus* infection, has not been studied. The present study is the first report to investigate the role of Th9 cells in human hydatidosis.

Results in this study show that the percentage of Th9 cells and IL-9 cytokine level was significantly increased in CE patients. After treatments of surgery and albendazole, the percentage of Th9 cells and IL-9 cytokine level was significantly decreased. These results indicate that decreased levels of Th9 cells and IL-9 might be used as prognostic factors for CE patients. However, the correlation between Th9/IL-9 profile and prognosis of CE patients still needs further investigation. It has been reported that IL-4 combined TGF-*β*, promoting naïve CD4^+^T cell differentiated into Th9 cells. In our study, cytokines levels of IL-4, TGF-*β*, and IL-10 in the serum of CE patients before treatment were increased, whereas they decreased after treatment. Researches have shown that levels of IL-4, IL-10, and TGF-*β* were all obviously increased after* Echinococcus* infection both in CE patients and the experimental mouse model [7, 31]. Results of these studies suggest that IL-4/IL-10 impairs the Th1 protective response and allows the parasite to survive in hydatid patients [32–34]. Our data were consistent with the above mentioned reports and further indicate that the complex cytokines network such as IL-9, IL-4, IL-10, and TGF-*β* might be involved in the regulation of Th9 cell differentiation in CE patients. Th9 and IL-9 were involved in Eg infection and might play potentially beneficial roles in the growth of Eg at the late stage after infection.

It is reported that transcription factors of PU.1, STAT6, IRF-4, and GATA-3 are involved in the differentiation of Th9 cells [9, 10, 15, 16, 30, 35]. PU.1 is a key transcription factor in the development of Th9 cells. GATA-3, a down-steam effector of STAT6, and IRF-4 also play an important role in Th9 cell differentiation [8, 36]. In this study, we also investigated the mRNA expression levels of PU.1, GATA-3, and IRF-4 in peripheral blood. Expression levels of PU.1 and GATA-3 mRNA were increased in CE patients and decreased significantly in PCE group. The changes in IRF-4 mRNA level of CE patients before and after treatment were not significant. Our data indicate that Eg antigen may stimulate the activation of PU.1 and GATA-3, but not IRF-4, and that PU.1 and GATA-3 may regulate the differentiation and proliferation of Th9 cells during Eg infection. In addition, we found that expression levels of IL-9 mRNA were significantly increased in CE patients before treatment. After treatments of surgery in combination with albendazole, despite the decrease in PU.1 and GATA-3 levels, the level of IL-9 mRNA was not significantly changed. Interestingly, percentages of Th9 cells (which secret IL-9 cytokine) and levels of IL-9 cytokine in peripheral blood were also significantly decreased after treatment. This inconsistency in changes of IL-9 mRNA and IL-9 cytokine might be due to other IL-9 transcription factors that kicked in or the delayed responses at the transcription level. The exact mechanism still needs further investigation.

In the present study, we have found that the numbers of Th9 cells, Th2 cells, and Treg cells were obviously increased, whereas the numbers of Th1 and Th17 cells did not change much in CE patients. It has been reported that several Th subsets, such as Th1/Th2 cells [5–7] and Th17/Treg cells [19], are involved in the regulating the immune response of Echinococcosis. Th1 and Th2 cells mainly exert their biological effects through secreting the cytokines of IFN-*γ* and IL-4. CE and AE patients show an upregulation of Th2 immune response at the late stage of infection, which has been shown in the liver lesions locally and in circulating lymphocytes and results in more rapid metacestode growth [37, 38]. There is, however, evidence that cellular immunity and Th1 cytokines also play a role, by controlling totally parasite growth in some individuals and in limiting the size of the lesions in the patients with the diseases [5]. Treg cells inhibit the immune response of effector T cells by secreting large amounts of IL-10 and TGF-*β*, which play critical roles of immune evasion in human parasitic infection [37–39]. Clearly, Th1/Th2 imbalance plays an important role in controlling the immunopathogenesis. Our data showed that the Th9, Th2, and Treg ratios in CE patients were increased more effectively than Th1 and Th17 cells.

For the correlation analysis, the number of Th9 cells in CE patient was positively correlated with that of Th2 cells, but not with that of Th1, Tregs, or Th17 cells. Moreover, IL-9 was also positively correlated with IL-4. Our correlation results suggest that Th9 cells may share mechanistic features of Th2 cells, as they have been associated with* Echinococcus* infection. Thus besides the imbalance of Th1/Th2 cells, there also might be Th1/Th9 imbalance in CE patients. It is reported that progressive growth of Eg during late stages of infection resulted in the persistence of the Th2 shift [40, 41]. Therefore, as Th2 is predominant at the late stage of Eg infection, Th9/IL-9 may associate with the occurrence and development of Eg infection. Levels of IL-4, IL-10, and TGF-*β* were significantly decreased in PCE group, suggesting that without stimulation of Eg, the immune response and regulation may be changed in the CE patients. IL-9 can affect different inflammatory cells, such as T cells, B lymphocytes, mast cells, and neutrophils, leading to proinflammatory effects [15, 42, 43]. However, the exact underlying mechanism of IL-9's role in the Eg inflammation response needs further study.

Overall, we reported for the first time that the Th9 cells and IL-9 expression were upregulated in CE patients but decreased after surgical and chemotherapy recovery. Our data suggest that Th9/IL-9 may be involved in regulating the immune response in the infection with* Echinococcus*. Th9/IL-9 may contribute to the process of Eg infection.

## Figures and Tables

**Table 1:** Clinical data of the healthy control (HC) and patients with liver echinococcosis before and after treatment.

Characteristics	HC (*n* = 25)	CE (*n* = 27)	PCE (*n* = 27)
Age (year)			
0–18	0	0	0
19–37	20	23	23
38–56	5	3	3
>57	0	1	1
Sex ratio			
Male : female	14 : 11	17 : 10	17 : 10
Location			
Right segment	—	18	—
Left segment	—	7	—
Right and lest segment	—	2	—
Lesions			
Diameter (cm)	—	8.7 ± 3.1	—
Residence			
Urban : rural	22 : 3	2 : 25	2 : 25
Races			
Han	14	15	15
Uighur	6	2	2
Kazakh	3	7	7
Other	2	3	3
WHO-IWGE classification			
CE1	—	5	—
CE2	—	14	—
CE3	—	7	—
CE4	—	1	—
CE5	—	0	—
Administered therapies			
Surgical removal	—	27	—
Albendazole treatment	—	27	—
Relapsing patients	—	0	0

*Note*. Values are expressed as mean ± SD or number. HC: healthy control; CE: cystic echinococcosis; —: not determined.

**Table 2:** Primers and cycling parameters for real time RT-PCR detection of PU.1 and IL-9.

Genes	Gene bank accession	Primer sequences (5′ to 3′)	Tm (°C)	Expected product sizes
PU.1	NM_003120.2	F: GGAGCCCGGCTGGATGTTAC	60.04	79 bp
		R: CACCAGGTCTTCTGATGGCTGA	60.03	
IL-9	NM_000590.1	F: CTCTAGCAGTCCACTTCACCAA	59.96	112 bp
		R: ACAGCATGGGTCTGTCTTCT	58.64	
GATA-3	NM_001002295.1	F: AGCATGAAGCTGGAGTCGTC	60.6	141 bp
		R: ACAGTTCACACACTCCCTGC	58.7	
IRF-4	NM_002460	F: GACCCGCAGATGTCCATGAG	60.53	82 bp
		R: TGTAGTTGTGAACCTGCTGGG	60.2	
*β*-Actin	NM_001101.3	F: TAGGCGGACTGTTACTGAGC	59.18	233 bp
		R: TGCTCCAACCAACTGCTGTC	60.82	

*Note*. F: forward; R: reverse.
